# Diurnal Oscillations of Soybean Circadian Clock and Drought Responsive Genes

**DOI:** 10.1371/journal.pone.0086402

**Published:** 2014-01-27

**Authors:** Juliana Marcolino-Gomes, Fabiana Aparecida Rodrigues, Renata Fuganti-Pagliarini, Claire Bendix, Thiago Jonas Nakayama, Brandon Celaya, Hugo Bruno Correa Molinari, Maria Cristina Neves de Oliveira, Frank G. Harmon, Alexandre Nepomuceno

**Affiliations:** 1 Embrapa Soybean, Brazilian Agricultural Research Corporation, Londrina, Paraná, Brazil; 2 Department of Biology, State University of Londrina, Londrina, Paraná, Brazil; 3 Plant Gene Expression Center, ARS/USDA, Albany, California, USA and Department of Plant and Microbial Biology, University of California-Berkeley, Berkeley, California, USA; 4 Department of Crop Science, Federal University of Viçosa, Viçosa, Minas Gerais, Brazil; 5 Embrapa LABEX US Plant Biotechnology, Plant Gene Expression Center-ARS/USDA, Albany, California, United States of America; McGill University, Canada

## Abstract

Rhythms produced by the endogenous circadian clock play a critical role in allowing plants to respond and adapt to the environment. While there is a well-established regulatory link between the circadian clock and responses to abiotic stress in model plants, little is known of the circadian system in crop species like soybean. This study examines how drought impacts diurnal oscillation of both drought responsive and circadian clock genes in soybean. Drought stress induced marked changes in gene expression of several circadian clock-like components, such as *LCL1*-, *GmELF4*- and *PRR*-like genes, which had reduced expression in stressed plants. The same conditions produced a phase advance of expression for the *GmTOC1*-like, *GmLUX*-like and *GmPRR7*-like genes. Similarly, the rhythmic expression pattern of the soybean drought-responsive genes *DREB-*, *bZIP-*, *GOLS*-, *RAB18*- and *Remorin*-like changed significantly after plant exposure to drought. *In silico* analysis of promoter regions of these genes revealed the presence of cis-elements associated both with stress and circadian clock regulation. Furthermore, some soybean genes with upstream ABRE elements were responsive to abscisic acid treatment. Our results indicate that some connection between the drought response and the circadian clock may exist in soybean since (i) drought stress affects gene expression of circadian clock components and (ii) several stress responsive genes display diurnal oscillation in soybeans.

## Introduction

Plants are subjected to diurnal oscillations due the planet movement around its axis, which generates light and temperature variations. In addition to the normal day/night variations, plants are subject to other environmental variations via biotic and abiotic stresses. Drought-induced water deficit greatly affects plant development, and in crop species this is damaging for agronomic productivity. Drought stress leads to a number of molecular and physiological changes in plants that protect against water deficit. Signal transduction molecules play important roles in this process by mediating the transmission of the stress signals via complex signal transduction pathways. In *Arabidopsis*, the molecular drought response mechanism can be divided into abscisic acid (ABA)-dependent and ABA-independent pathways [1]. The ABA-dependent signal transduction pathway comprises the ABA-bound pyrabactin resistance/regulatory component of ABA receptor (PYR/RCAR) proteins, type 2C protein phosphatases (PP2C), and SNF1-related kinases (SnRK2) [2]. Additionally, the H subunit of the magnesium-protoporphyrin IX chelatase protein (ABAR/CHLH/GUN5) has also been described as ABA receptor in *Arabidopsis* under stress conditions [3]. ABA-mediated signal transduction leads to the activation of transcription factors, such as basic leucine zipper (bZIP) proteins [4]. In contrast, the ABA-independent pathway involves ethylene signaling and the participation of transcription factors, primarily from the ethylene-responsive factor (ERF) and C-repeat-binding factor/dehydration-responsive element-binding (CBF/DREB) subfamilies [1], [5]. In both the ABA-dependent and ABA-independent pathways, transcription factors bind to specific *cis*-elements and induce several stress-responsive genes that encode different protein classes, including galactinol synthase (a key enzyme for the biosynthesis of the osmoprotectant molecule raffinose) [6], RAB18 (a protein involved in membrane vesicle transport) [7], Remorins (membrane structural proteins) [8], and peroxidases (ROS-scavenging proteins) [9].

The circadian clock is an endogenous timer that plays a key role in the coordination of plant biological activities with diurnal variations, conferring adaptive advantages to organisms. In this system, environmental cues like light and temperature play important role in entrain the circadian clock responses. Recent studies have demonstrated a correlation between the circadian clock and plant responses to drought, suggesting a close connection between both pathways [10], [11]. The more recent existing model of the circadian clock in plants comprises interlocked feedback loops, which includes a ring of three sequential negative steps: (a)the inhibition of evening complex (EC) genes (ELF3, LUX, and ELF4) by the rise of LHY/CCA1 in the late night, (b) the inhibition of PRR genes by EC in the early night, and (c) the inhibition of LHY/CCA1 by PRRs in the day [12]. The evening complex is suggested to represent the structure of the evening loop [12]. The two partially redundant morning Myb-like transcription factors CCA1 and LHY regulate the expression of TOC1 and GI. TOC1/PRR1 is a member of the clock-specific transcription factor family of pseudo-response regulator (PRR) proteins, and GI is a vascular plant-specific protein with a poorly understood molecular function. CCA1 and LHY reach peak levels in the morning to repress the daytime expression of TOC1 and GI. CCA1 and LHY directly bind to the TOC1 promoter at a 9 bp target sequence referred to as the evening element (EE; AAATATCT), and this interaction suppresses transcription at this promoter [13], [14]. Recently, Pokhilko and colleagues extended the inhibitory action of LHY/CCA1 to all evening genes (TOC1, LUX, ELF4, ELF3 and GI) [12]. TOC1 appears to repress CCA1 and LHY expression inside the morning loop [12], [15], while the EC indirectly activate CCA1/LHY expression, by suppressing the expression of CCA1/LHY’s repressors [12], [16]–[18]. Two additional loops maintain the adequate expression of CCA1/LHY and TOC1. In the morning, PRR5, PRR7 and PRR9 are activated through the activity of CCA1/LHY, and in turn PRR5, PRR7 and PRR9 feedback repress the expression of CCA1/LHY [19], [20]. In a posttranslational loop, the F-box protein ZTL targets TOC1 for degradation [21]. GI controls the activity of ZTL, and the physical interaction between GI and ZTL (light dependent) stabilizes both ZTL and TOC1 expression during the day [21]. According to the most recent circadian clock model in Arabidopsis, GI increases TOC1 expression by the inhibition of the EC, which is a negative regulator of TOC1 expression [12]. Together, the three interlocking feedback loops ensure that the clock produces robust and accurate rhythms.

Although our present knowledge of the circadian clock suggests conservation among plant species, studies of clock function and molecular architecture in plants other than *Arabidopsis* are limited [22]. Soybean is one of the most well-studied crops in terms of its genetics and related molecular behavior under many circumstances; however, there is a lack of information concerning the behavior of the soybean circadian oscillator. Some orthologs to the *Arabidopsis* circadian clock genes, have been identified in the soybean genome and have been shown to oscillate in a manner similar to that in *Arabidopsis* in controlled situations [23]–[26]. Previous studies in Arabidopsis have shown connections between the plant responses to abiotic stresses (eg. heat, cold and drought) and the diurnal oscillations or the circadian clock [10], [11], [27], [28]. However, to date the behavior of the soybean clock components in response to environmental stresses, like drought, was not investigated. Environmental cues like light, temperature and abiotic stresses can act as inputs that modulate the circadian clock, ensuring the precise synchronization of important plant molecular processes [29]. For crop species, such as soybean, flowering is a key component of productivity, whereby precise synchronization using environmental cues maximizes the number of flowers and pods produced and, consequently, increases the yield. Understand the impact of drought on the circadian clock components is of great interest, once the drought imposition can act as an environmental cue to the clock and the processes it controls.

## Materials and Methods

### 1. Plant material, growth conditions, and treatment application

The seeds from plants of the BR16 genotype, which exhibit drought-sensitive characteristics [30], were cultivated in peat pots (Jiffy) with Supersoil® (Scotts Miracle-Gro Company, Marysville, Ohio, USA). The plants were grown in growth chambers set to simulate environmental conditions: 14 h light/10 h night cycles, with 500 µmol m^–2^s^–1^ of white light (provided by cool white fluorescent bulbs), with 28°C/20°C temperature cycles during the light and the dark period, respectively. Fifteen days after germination, when the plants reached the V2 developmental stage [31], water was withheld in the stress treatments to induce a water deficit. The soil moisture was calculated by the gravimetric humidity (GH), which corresponds to the percentage of water in the soil in relation to the dry weight of the soil. The volume of irrigation was adjusted to 70% (GH) (near field capacity) for the unstressed treatment, 30% GH for the moderate stress treatment, and 15% GH for the severe stress treatment. The pots were weighed twice a day, and water was added to maintain the treatments at the desired GH values. Sampling was initiated when 30% and 15% GH were obtained for the moderate and severe stress treatments, after 3 and 5 days of water withholding, respectively. To overcome the differences due developmental stage differences between plants from the 3^rd^ and 5^th^ days of water withholding, control plants (maintained at 70% GH) were collect for each day. In other words, control and stressed plants were age-matched. Fully expanded V1 leaves were collected from the 3 plants in each treatment at 4 h intervals from the time the lights came on (Zeitgeiber Time (ZT) 0), and were immediately frozen in liquid N_2_ and stored at −80°C until further use. The samples obtained in the dark were collected with the aid of a small green LED light (PhotonLight.com).

For the ABA treatment, the BR16 plants were grown under the same conditions as described above. Fifteen days after germination (V2 developmental stage, [31]), ABA (MP Biomedicals; Santa Ana, California, USA) was sprayed directly onto the plants using 4.5 mL of a mixture of 100 mM ABA and 0.01% (v/v) Triton X-100 in water, in accordance with the methods of Legnaioli et al. (2009) [10]. The untreated plants received a mock treatment of 0.01% (v/v) Triton X-100 in water. The ABA solution was applied at ZT3 (11:00 am), and the samples were collected at the indicated time points and analyzed.

### 2. Real-time qPCR analysis of gene expression

The gene expression was monitored for the samples subjected to moderate and severe stress (30% and 15%GH, respectively) compared to unstressed treatment situation (70% GH), using quantitative real-time PCR (qPCR). A similar approach was used to compare samples subjected to 100 mM ABA treatment and the control plants (not treated with ABA). All of the experiments were completed with three biological replicates, consisting of two plants collected together and pooled. Each replicate tissue set was ground to a fine powder in liquid nitrogen, and the total RNA was isolated using the Concert Plant RNA Reagent (Life Technologies, Grand Island, NY, USA) according to the manufacturer’s instructions. The contaminating DNA in the total RNA was removed using the Turbo DNA-free kit according to the manufacturer’s instructions (Ambion by Life Technologies, Grand Island, NY, USA). The high-quality total RNA was used to analyze the transcripts in each treatment at 4 h intervals from the time the lights came on (Zeitgeiber Time (ZT) 0). The first-strand cDNA was generated using the Maxima Universal first-strand cDNA synthesis kit (Fermentas/ Thermo Fisher Scientific Inc., Waltham, MA, USA), according to the manufacturer’s instructions and diluted five times with water, and 2 µL was used for the qPCR using a CFX Real-Time PCR Detection System (Bio-Rad, Hercules, CA, USA), with in two technical replicates. The qPCR reactions contained 1X EvaGreen Dye (Biotium, Hayward, CA, USA), 1X ExTaq buffer (Clontech, Mountain View, CA, USA), 200 µM each dNTP (New England Biolabs, Ipswich, MA, USA), 300 µM each primer, 0.05 mg/mL BSA (New England Biolabs, Ipswich, MA, USA), 0.1% Tween-20, and 5% DMSO mix.

The normalized cycle threshold (Ct) values were calculated based on the geometric average of two endogenous genes (Elongation factor 1-β, Glyma13g04050, and β-actin, Glyma15g05570) previously identified as stably expressed under many developmental stages and stress situations [32], [33]. The normalized expression (NE) was calculated using the following formula: NE = 2∧^-(ΔCt)^, where ΔCt =  Ct experimental – Ct normalizer (n). The primers for the *Glycine max*-like clock genes were designed using soybean Glyma ortholog sequences from Phytozome *G. max* v.1.0 (see Table 1). The soybean drought-responsive genes were chosen according to previous work confirming the upregulation of these genes under conditions of dehydration [34], [35]. The primer sequences (Table 1) were designed using the PrimerQuest tool (Integrated DNA Technologies, Coralville, IA, USA), employing the sequence from the 3′ untranslated region with the default settings. Gene expression was evaluated in 3 independent biological replicates, each one evaluated in 2 technical replicates. To compare gene expression between control and stressed/treated plants along a time course we performed statistical analysis using ΔCt values, as described by Yuan and colleges [36]. After performing descriptive and exploratory data analysis, the effects of water regimes (control, moderate and severe drought) or ABA treatment, day time (ZT0 to ZT20), and their interactions on determined variables were analyzed using ANOVA. When results from the overall significance test led to rejection of the null hypothesis, Duncan’s multiple range test for multiple comparisons among groups tests (5%) was performed. The above analyses were conducted using SAS 9.2 software (SAS Institute, Cary, NC).

**Table 1:** *Arabidopsis* and soybean ortholog genes.

Gene name	Arabidopsis gene	Soybean ortholog gene	Soybean name	TBLASTN (e-value)	Identity (%)	Forward primer (5'->3')	Reverse primer (5'->3')
TOC1	AT5G61380	Glyma04g33110	GmTOC1-like	5.2e-52	40.3	TGACATAAGGATGAAGGGCCAACC	TGAGGGCGCATATTGGATCAACAC
PRR7	AT5G02810	Glyma10g05520	GmPRR7-like	8.3e-38	59.1	GGCAACAATTCTGGCACCACCTAA	GCAGCTGATGCTTCATGTTGTCAG
PRR9	AT2G46790	Glyma06g14150	GmPRR9-like	2.6e-36	67.7	CCCGAATCCTTAAATACCAGAAGCAC	CACGATCTACAGAAAGGGCAAATG
PRR3	AT5G60100	Glyma11g15580	GmPRR3-like	4.3e-36	89.1	TGATGTCATCTCATGATTCTATGGGT	ACTCACACTGTGGCATCTTCTCCA
CCA1	AT2G46830	Glyma07g05410	GmLCL1-like	1.6e-21	40.3	ACCATAGGGCTTGGACAAGGAAAG	ACCTTGATTGTTGCTCGCTCCAAC
LHY	AT1G01060	Glyma07g05410	GmLCL1-like	1.6e-21	40.3	ACCATAGGGCTTGGACAAGGAAAG	ACCTTGATTGTTGCTCGCTCCAAC
ZTL	AT5G57360	Glyma09g06220	GmZTL-like	0	88.7	GCATGCTGTAGCAAGGGAAATGCT	CTGACCAGAGCAATACTCGTCAAG
GI	AT1G22770	Glyma20g30980	GmGI-like	0	82.2	GTGGCAGATGGCCTTTCAAACCTT	CGGACATGTGCACTTGGATGAGAA
LUX	AT3G46640	Glyma12g06410	GmLUX-like	5.6e-37	90.2	GAACCTAAGGTCAGCAGCAATCAC	TCAATTCGATCTCCTGCCAAATGC
ELF4	AT2G40080	Glyma18g03130	GmELF4-like	4.1e-26	73.1	ATTCAGCAGGTGAACGAGAACCAG	ACAACCTTGGAGATGTTGCCGTTG
CHE	AT5G08330	Glyma20g00350	GmCHE-like	3.2e-28	59.9	TATTGTGTTTGTCGGTGGGTGGGT	AGTCCTTCTCCTTGTCCACACACA
JUMONJI	AT3G20810	Glyma11g13910	GmJumonji-like	1.3e-36	58.5	TTTGGCACTCGTTGTCACTACACG	TACTGTTCGGGACTGCGTTTCACA
ABAR	AT5G13630	Glyma19g32070	GmABAR-like	0	88.7	AGAGAAGAGCAGCATCCTTCA	TTCAGAACTGCACAAACGAGA
REMORIN	AT2G41870	Glyma19g32280	GmRemorin-like	8.6e-33	47.1	TGGATTGCAGTAAGCAGCAC	AGCGTGACACCACTTATCACA
GOLS	AT2G47180	Glyma19g40680	GmGOLS-like	2.0e-73	79.4	ACGGGGAAGGAAGAGAACAT	TGCACTCATCAATGGCTTGT
DREB1	AT1G46768	Glyma14g09320	GmDREB1-like	5.5e-39	55.9	GATGATGATGCCTCGGAGTTG	CGGAAAAACAAGAAAAGGGATATATC
DREB2	AT4G39780	Glyma05g31370	GmDREB2-like	2.0e-46	47.5	GGCTGCTTCTGCAATGGATT	GACCACTACGACCCTCTCTGATTT
DREB3	AT1G22190	Glyma13g01930	GmDREB3-like	1.2e-41	44.3	TTGCTTATTGGCTATTCGATGGT	TCCATGGCCAAGCAAGAAA
RAB18	AT5G66400	Glyma09g31740	GmRAB18-like	1.0e-12	36	CAACTGGTGGCACTGGTTATGG	TGGTCATGCTGACGATGTTCCT
bZIP	AT3G19290	Glyma02g14880	GmbZIP	5.4e-45	42.8	TAATGGGAATGGGAATTTGGG	GTTGGTGTTGGTGTTGGTGTTGTG
PP2C	AT1G07160	Glyma14g37480	GmPP2C-like	2.7e-80	70.4	GCTATGTTGATTTATGCCGTGGTG	ACTTTGGTCTCAGGCTCTGCTGTCA
SnRK2	AT4G33950	Glyma02g15330	GmSnRK2-like	6.7e-158	92	CAAAGTGATCTCATGGATGGGA	TGCTATCTAAGTCAAGGTCAGGATC

The **Arabidopsis gene** identification using the TAIR database and the **soybean** **orthologous** gene identification using the Phytozome database are shown.

The **forward** and **reverse primer** sequences correspond to the oligonucleotides used to amplify the soybean orthologs.

**The TBLASTN e-value** and **Identity** correrspond to the local alignament between the Arabidospsis protein and the soybean translated genome at Phytozome database.

### 3. Gene expression analysis by RNA-seq

The soybean transcriptome was analyzed by RNA-seq in samples subjected to moderate stress (30% GH) compared to unstressed treatment situation (70% GH). After DNase treatment (Life Technologies, Grand Island, NY, USA), high-quality total RNA was used to analyze the transcripts for each time point: 8, 12, 16, 20, 24, and 4 h. Bulks of leaves from two plants were used in the RNA extraction to compose one replication. Three replications for each time point/treatment were sequenced. The RNA-seq libraries were built using the Nugen-Ovation® kit according to the manufacturer’s instructions (NuGEN Technologies Inc., San Carlos, CA, USA). The libraries obtained were subjected to sequencing by Illumina HiSeq2000 (Illumina, San Diego, CA, USA). Mapping of the reads was performed with the Soybean genome (Phytosome Glycine max v1.1) using the GeneSifter platform (http://www.geospiza.com/Products/AnalysisEdition.shtml). To compare gene expression between different times and conditions, we log_2_-transformed the normalized reads per mapped million (RPM) value. Data were analyzed using ANOVA to evaluate the effects of water regimes (control and moderate drought), time point (ZT0 to ZT20), and their interactions. We performed Tukey’s HSD multiple comparison tests (95% family-wise confidence level) to show the interactions between water regimes (control and moderate drought) and time points, whenever these interactions were significant in ANOVA analyses. The RNA-seq analyses were performed using the GeneSifter Analysis Edition platform (GSAE; a registered trademark of Geospiza, Inc.) [37].

### 4. Identification of soybean circadian clock genes

To identify homologs of the *Arabidopsis* circadian clock genes in the soybean genome, the amino acid sequences of the corresponding *Arabidopsis* proteins were used as queries in BLAST searches (TBLASTN tool) [38] in the *G. max* genome v1.0 using the Phytozome database (http://phytozome.net/soybean). The most similar sequences were selected on the basis of whether they had alignment e-values close to 0, using the cutoff e-value 1e-20. The phylogenetic tree construction was based on the alignment of the amino acid sequences using ClustalW and the generation of trees using the Neighbor-Joining method [39] in the MEGA 5.0 software [40]. The percentage of replicate trees in which the associated taxa were clustered together in the bootstrap test (1000 replicates) [41] are shown next to the branches. The tree was drawn to scale, with the branch lengths in the same units as those of the evolutionary distances used to infer the phylogenetic tree. The evolutionary distances were computed using the Poisson correction method [42] and are presented as the number of amino acid substitutions per site. All of the ambiguous positions were removed for each sequence pair (pairwise deletion) [43].

### 5. Identification of soybean circadian clock genes and cis-elements in the promoter regions of circadian clock and drought-responsive genes

The putative *cis*-regulatory elements in the promoter regions of the soybean genes were identified using a suite of Genomatix programs (http://www.genomatix.de, Genomatix Software, Munich, Germany) [44] (Tables S1 and S2). First, the Gene2promotor component was used to define the promoter region, which encompassed 500 bp upstream of the transcriptional start site and 100 bp downstream of the transcriptional start site into the presumed 5′ untranslated region. Second, each 601 bp sequence was examined for putative target sequences of well-established plant transcription factors using MatInspector Version 4.3 [44]. The MatInspector program search for *cis*-elements was based on a “Matrix” consisting of a position weight matrix, a conservation profile (conservation index vector), and a core region for a set of training sequences. The “matrix similarity score” reflects the similarity between the obtained sequence and the matrix sequence, whereby a value of one corresponds to sequences with the most conserved nucleotide at each position in the matrix. Mismatches in the highly conserved positions of the matrix decrease the matrix similarity more than mismatches in the less-conserved regions [44], [45]. We selected a threshold matrix similarity of >0.80 based on previous studies using sequence-training sets, which indicates that a consistent match to the matrix has a similarity of >0.80 [44].

## Results and Discussion

### 1. Drought stress induces significant changes in the diurnal expression of putative circadian clock genes

To characterize the effects of drought stress on the transcriptional regulatory networks of the soybean circadian clock effectors, genes encoding probable circadian clock components were identified through in-house alignments of the amino acid sequences of core *Arabidopsis* circadian clock proteins to the *G. max* genome. We identified potential soybean homologs of TOC1, CCA1/LHY, LUX, ELF4, CHE, GI, ZTL, and several PRR-like proteins (Table 1). The soybean genome encodes at least two *LHY* and *CCA1-*like homologs named *LCL1* and *LCL2* (*LHY/CCA1-*like 1 and 2), however it is not possible distinguish which gene (LCL1 or LCL2) corresponds to the Arabidopsis LHY or CCA1 [23]. In this study we evaluated specifically *GmLCL1* gene expression. The PRR proteins are highly similar in amino acid sequence, and the soybean genome is highly redundant [46]; therefore, a phylogenetic analysis was used to identify the most probable homologs for each PRR class (Fig. S1). In total, 11 genes encoding potential circadian clock genes were identified for our analysis (Table 1).

**Figure 1:**
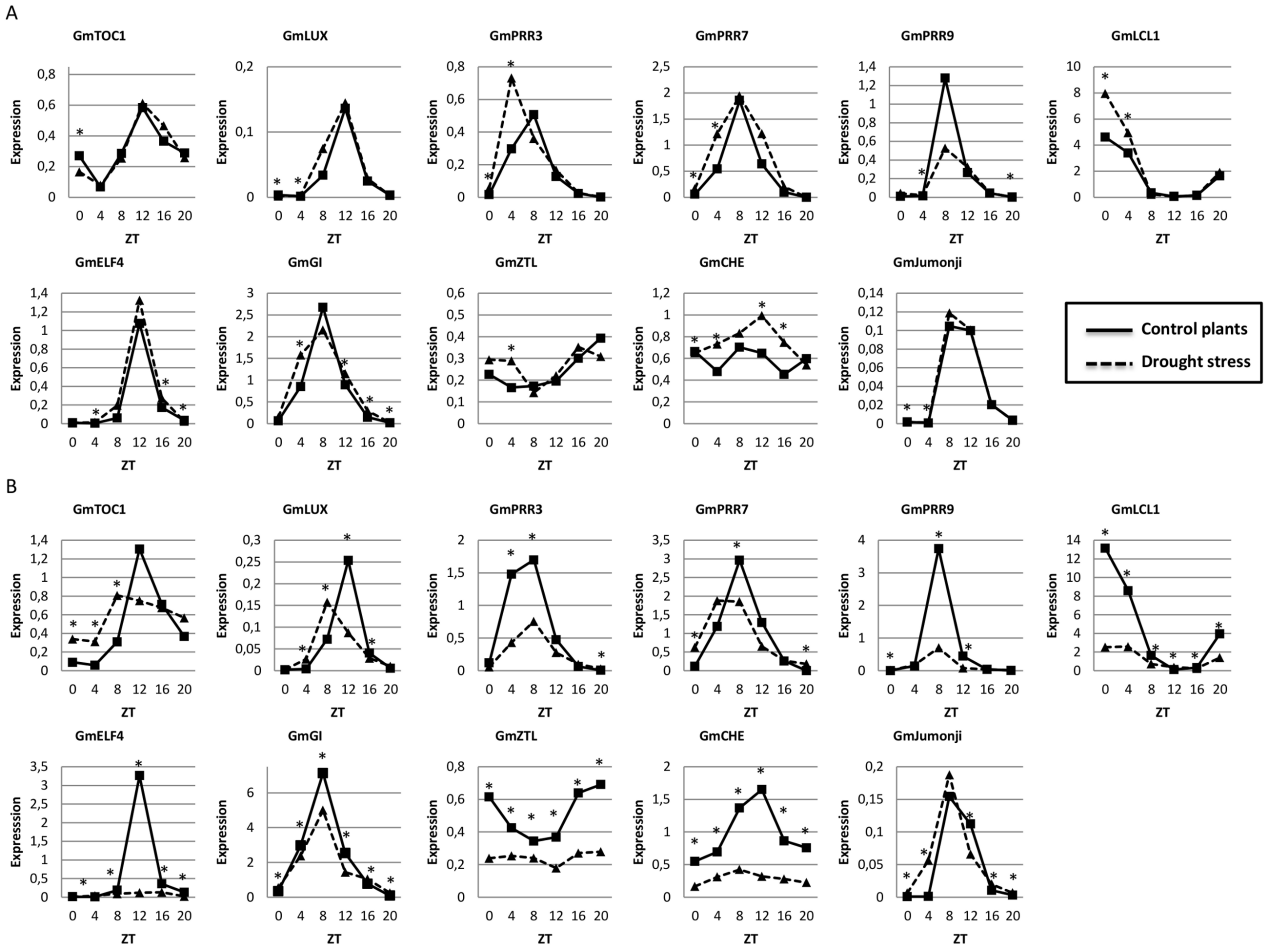
Drought affects the expression of some circadian clock genes in soybean. Gene expression data regards qPCR analysis of soybean leaves during moderate (A) and severe (B) drought stress. Expression axis represents normalized expression (NE) = 2∧^-(Ct experimental – Ctn)^. Collect time points are represented by ZT (Zeitgeiber Time) 0 to 20, starting from the time the lights came on ( ZT0) and proceeding with 4 h intervals until ZT20. For easy viewing, asterisks represent significant differences between control and stressed plants in each time point (Duncan’s test 5%, *time -treatment* interaction). The ANOVA and the complete Duncan’s test results are presented in Table S3.

**Figure 2:**
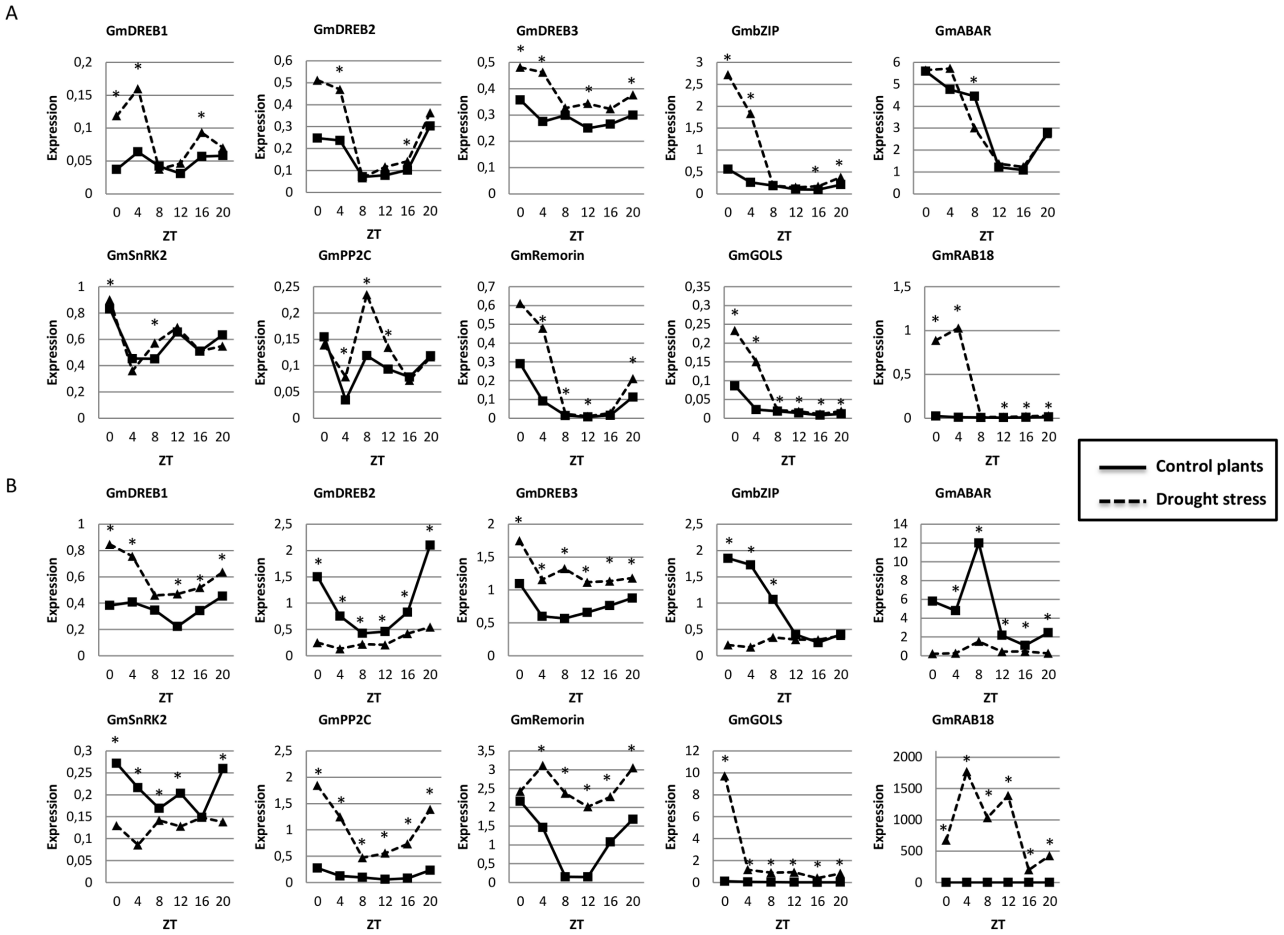
Drought-responsive genes in soybean exhibit diurnal regulation, and the expression pattern is modified under drought stress. Gene expression data regards qPCR analysis of soybean leaves during moderate (A) and severe (B) drought. Expression axis represents normalized expression (NE) = 2∧^-(Ct experimental – Ctn)^. Collect time points are represented by ZT (Zeitgeiber Time) 0 to 20, starting from the time the lights came on ( ZT0) and proceeding with 4 h intervals until ZT20. For easy viewing, asterisks represent significant differences between control and stressed plants in each time point (Duncan’ test 5%, *time -treatment* interaction). The ANOVA and the complete Duncan’s tests results can be found in Table S3.

The great plasticity exhibited by plant responses to drought calls for studies that simulate field conditions in order to obtain information on the plants responses under more natural conditions. A recent study showed that molecular analyses under constant environmental conditions (eg. constant light, temperature) may give partial or misleading indications of the plant responses to the natural fluctuating conditions [47]. Therefore, studies showed that light and temperature cycles entrain the clock and thereby ensure appropriate phasing of circadian rhythms [18], [48]. In this context, to assess the impact of drought stress on the soybean circadian clock components, the expression profiles for each candidate soybean circadian clock gene were determined in V2-stage plants under simulated field light (14h light/10h dark) and temperature (28°C/20°C) cycles, over a 24 h time course under well-watered conditions (control) or one of two water-limited (stress) conditions. Prior to the implementation of the stress treatments, all of the plants were maintained under well-watered conditions, which corresponded to soil at 70% GH. After reaching the V2 stage of development, in accordance with the definition of previous study [31], irrigation was removed for the water-limited plants. The moderate stress condition was obtained when the soil achieved 30% GH, corresponding to 3 days of water withholding, whereas the severe stress treatment corresponded to a GH of 15%, achieved with 5 days of water withholding. To overcome possible differences in gene expression due disparity in plants developmental stages we compared age-matched stress and control plants, since authors have demonstrated changes in gene expression between plants in different developmental stages [49], [50]. As we can see in Figures 1 and 2, for some genes we observed differences in the expression of control plants from the moderate and severe stress, which points the importance of using age-matched plants for comparisons between control and stress.

To compare the qPCR gene expression between control and stressed plants we performed ANOVA statistical analysis, followed by Duncan’s test. In this analysis, we used the ΔCt values for treatment and control samples. This approach ensure the adjustment of additive effects of concentration, gene, and replicate variations since the ΔCt values are obtained subtracting Ct number of target gene from that of reference/normalizer genes[36]. Statistical results for ANOVA and Duncan’s tests are presented in Table S3.

Under normal well-watered conditions, the potential soybean circadian clock genes showed expression patterns compatible with the circadian oscillation described in literature for soybean [23], [24], [26] and Arabidopsis [12], [51]–[53]. On the other hand, the plants exposed to the moderate and severe drought stress conditions showed significant changes in the diurnal expression of several potential core circadian clock genes. The expression of *GmTOC1*-like under normal hydration conditions was compatible to previous circadian oscillation reports [23], [26] with transcript levels for this gene peaked during the transition from day to night at the time corresponding to ZT12 (Fig. 1). The moderate stress condition did not affect the timing of *GmTOC1-*like expression (Fig. 1A), whereas lower amplitude of *GmTOC1-*like peak expression was observed 4 hours earlier under severe stress conditions (Fig. 1B). The evening-expressed *GmLUX*-like also displayed advanced-phase expression under severe hydration stress (Fig. 1B). Similar to *TOC1*, the *LUX* gene expressed in the evening were reported to be direct regulatory targets of CCA1 and LHY in *Arabidopsis* [19], [54]. The advance in the peak expression of these evening genes was similar to that observed in *Arabidopsis* mutants lacking both the CCA1 and LHY functions [53]. The early phase expression observed in the *cca1 lhy* double mutants reflected the absence of the normal repression activities of these two transcription factors, suggesting that drought stress conditions modify the regulation of soybean *LHY/CCA1*-like gene expression.

To examine whether drought stress influenced the morning loop components of the soybean circadian clock, the expression of the *GmLCL1*-like gene was examined under conditions of drought stress. The expression of *GmLCL1*-like under conditions of normal hydration was comparable to that of its *Arabidopsis* ortholog (Fig. 1), with the peak expression of this gene being observed at dawn (ZT0). Strikingly, *GmLCL1*-like expression was substantially reduced in those plants experiencing severe dehydration stress (Fig. 1B). Therefore, the advancement of evening-phased gene expression under severe drought stress might reflect, at least in part, the loss of normal *GmLCL1*-like expression.

The lack of normal *GmLCL1*-like expression under drought conditions might also negatively affect the expression of *GmPRR9-*like and *GmPRR3-*like, as the transcript levels of these genes were markedly lower in the plants exposed to severe drought stress (Fig. 1B). The two *Arabidopsis* genes that participate in the morning loop, *PRR9* and *PRR7*, are directly upregulated through CCA1 and LHY [19]. Indeed, the genes we identified as *GmPRR9*-like and *GmPRR3*-like could potentially represent these components in the soybean clock system.

Although drought stress potentially influences Gm*LCL1-*like regulation, a potential contributor to the strong reduction in *LCL1* expression is the severe drought-induced loss of a *GmLCL1-*like activator. *GmELF4-*like is a putative activator of *GmLCL1-*like because *Arabidopsis ELF4* is required for the phytochrome-mediated light induction of *AtCCA1* and *AtLHY* [18]. Consistent with this idea, the *GmELF4-*like expression was low in the plants exposed to severe drought stress (Fig. 1). In *Arabidopsis*, ELF4 is repressed through the action of a multiprotein complex containing CCA1 and LHY and the transcription factors FAR1 and FHY3 [18], [55], [56]. FAR1 and FHY3 also activate ELF4 when CCA1 and LHY are absent [56]. Thus, severe drought stress could negatively affect the function of the soybean orthologs FAR1 and/or FHY3, resulting in limited *GmELF4-*like expression. Fig. S2 shows a proposed scheme for the effect of severe drought on the expression of *GmLCL*-like, *GmTOC1*-like, *GmLUX*-like, and *GmELF4*-like genes.

A general reduction in the amplitude of expression was observed after severe drought stress for most of the clock genes evaluated, including the *GmPRR3-*like, *GmPRR7-*like, *GmPRR9-*like, *GmGI-*like, *GmZTL-*like, and *GmCHE-*like genes (Fig. 1B). This observation is consistent with the effect of other abiotic stresses on the circadian rhythms in plants. Cold stress reduces the amplitude of cycles for clock components and reduces/disrupts the cycles of output genes in Arabidopsis, poplar, and chestnut [27], [57], [58].

Unlike the strong repression observed under the severe stress condition, the *GmLCL1*-like expression under moderate stress was slightly higher relative to that in the control plants at ZT0 and ZT4, with the most noticeable effect occurring at ZT0 and ZT4 (Fig. 1A). This increase in *LCL1* during early stress stages might be involved in the signaling and activation of drought response mechanisms. CCA1 directs the circadian regulation of the *CBF/DREB* genes in *Arabidopsis* [28], and CCA1 participates in the transcription factor complex that promotes the acute induction of CBF/DREB genes under cold stress [59]. A similar system might be induced in response to drought, as cross-talk between cold and dehydration response pathways is observed.

The *Jumonji* genes are upregulated by drought in soybean [34] and *Arachis hypogaea* [60]. The Jumonji transcription factors act as demethylases, controlling chromatin structure and, thus, gene expression [61]. The silencing of an *A. hypogaea Jumonji* ortholog in tobacco improved drought tolerance [60]. The *Arabidopsis JMJD5* (or *JMJ30*) gene shows circadian oscillation and acts as a regulator of period length [51]. It has been proposed that *JMJD5* and *AtTOC1* act in combination to control the circadian clock and that both genes are repressed through the activity of *AtCCA1* and *AtLHY* [62]. The soybean *GmJMJ-*like gene exhibited strong diurnal expression (Fig. 1). Severe drought treatment induced the upregulation of *GmJMJ-*like expression, particularly at ZT4, and the downregulation of *GmJMJ-*like expression at ZT12, relative to the control plants (Fig. 1B). Whether these changes in expression during drought stress might be important for the regulation of drought responses remains unknown. However, epigenetic mechanisms, such as DNA methylation and histone modification, play a crucial role in regulating gene expression during plant responses to environmental stress [63]–[66].

For crop species, such as soybean, corn or cotton, productivity is directly connected with flowering intensity/stability to maximize number of seeds produced. Clock genes expression is directly controlled by day length and temperature, interacting with other environmental factors (water status, soil fertility, presence of pathogens, etc) to establish flowering initiation, intensity and duration. Previous studies were able to increase soybean productivity by overexpressing the AtBBX32 transcription factor [67]. They demonstrated that the constitutive expression of AtBBX32 in soybean altered the abundance of transcript levels of the soybean clock genes *GmTOC1 and LHY-CCA1-like2* (*GmLCL2*), and by altering the abundance of circadian clock genes during the transition from dark to light, the timing of critical phases of reproductive development was altered [67]. Flower, pod, and seed number were increased, where the authors proposed that it was caused by changes in the timing of reproductive development in the transgenic soybean that lead to the increased duration of the pod and seed development period. Drought is well known to alter time of flowering in soybean where maintenance/abortion of flowers/seeds will depend on the intensity and duration of the stress. Our results are the first to show in soybean that water deficit alters the expression of circadian clock genes. Thus, in this context, an understanding of the means by which the circadian clock gene expression is altered by this environmental stress may give clues to how we can genetically manipulate the circadian clock to reduce yield losses during drought events.

### 2. The expression of drought responsive genes oscillates during the day

To evaluate the influence of the time of the day on the expression of soybean drought-related genes belonging to the ABA-dependent and ABA-independent drought response pathways and to determine the effect of drought on these genes temporal-dependent regulation, we analyzed the daily expression of 10 drought-responsive genes by qPCR and RNA-seq. The gene names and the respective orthologs in *Arabidopsis* and soybean are shown in Table 1.To compare the qPCR gene expression between control and stressed plants we also performed ANOVA statistical analysis, followed by Duncan’s test, similarly to the analysis of the circadian clock genes, previously described. Statistical results for ANOVA and Duncan’s tests are presented in Table S3.

**Figure 3:**
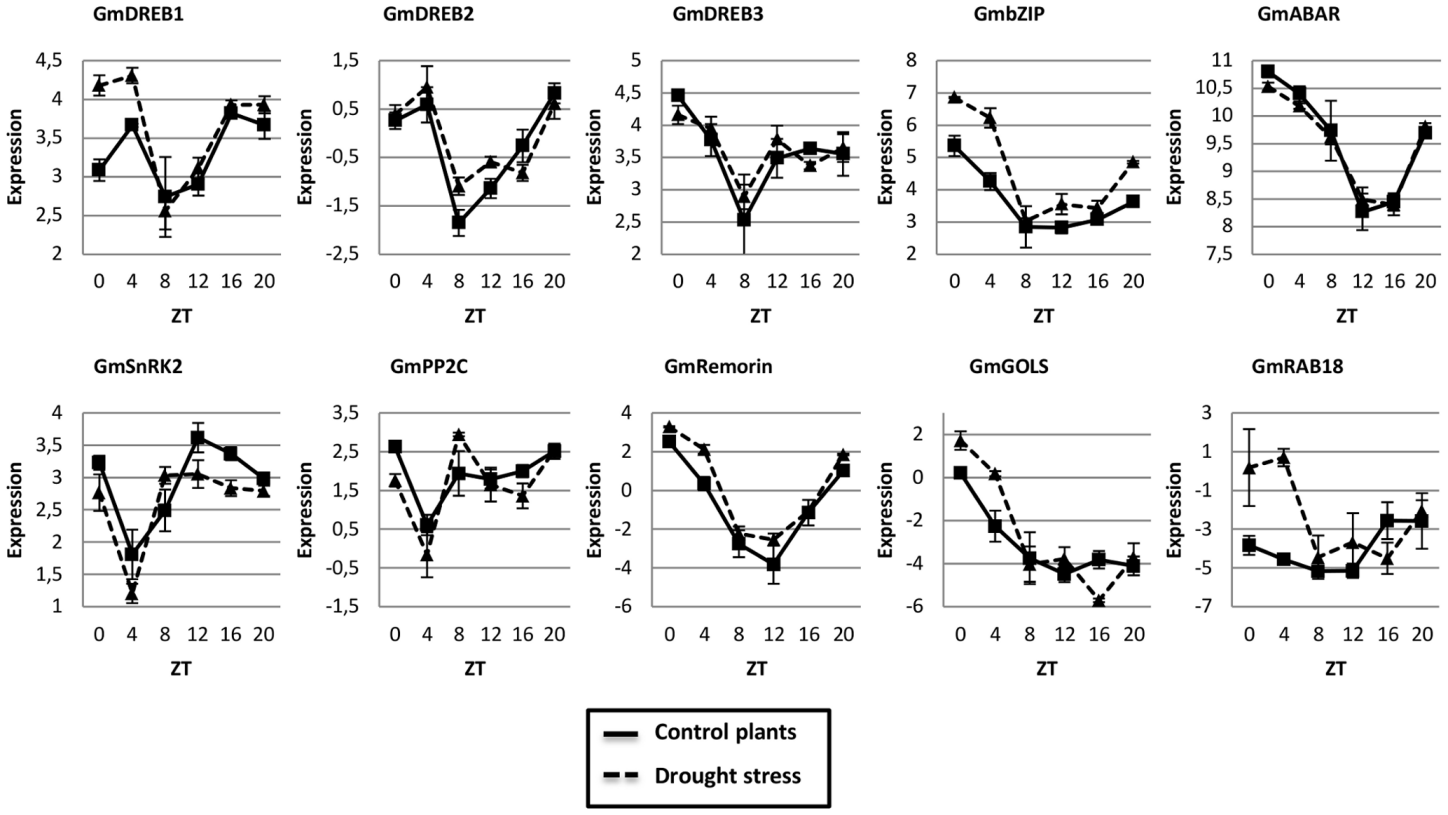
The diurnal oscillation of drought-responsive genes in soybean leaves during moderate stress is confirmed by RNA-seq analysis. Collect time points are represented by ZT (Zeitgeiber Time) 0 to 20, starting from the time the lights came on ( ZT0) and proceeding with 4 h intervals until ZT20. The error bars represent the standard error. The ANOVA and the Tukey HSD (95% family-wise confidence level) multiple comparison tests can be found in Table S4.

Several studies have demonstrated the circadian oscillation of stress and hormone-responsive genes in *Arabidopsis* [10], [11], [35], [68], [69]. Consistent with these data, the drought- and ABA-responsive soybean genes showed diurnal oscillations in gene expression on qPCR data (Fig. 2, Table S3). The diurnal oscillation of these genes could also be confirmed in RNA-seq data, where ANOVA statistical analysis, followed by Tukey’s test pointed differences in gene expression along the day (Fig. 3, Table S4).

In previous study, we demonstrated that some genes from the DREB subfamily displayed circadian oscillation under control and stressed conditions [70]. In the present study, we evaluate other DREB genes, to better understand the observed expression patterns and its relation with the soybean daily cycle. As expected, under control conditions, the expression of the *GmDREB-*like genes generally increased just before dawn and reached their peak expression between ZT0 and ZT4, in both, qPCR and RNA-seq analyses (Fig. 2 and 3). Generally, moderate stress induced the expression of *GmDREB1-*like, *GmDREB2-*like, and *GmDREB3-*like but had little effect on the timing of peak expression (Fig. 2). Interestingly, the morning-specific induction of these genes was associated with an increase in *GmLCL1*-like expression under the same treatment. The *Arabidopsis DREB* genes are positively regulated through the direct interaction of *CCA1* and *LHY* with the evening element motifs in the DREB gene promoters [28], [59]. Additionally, the promoter regions of the *GmDREB-*like genes contain putative evening elements (Table S2); thus, *GmLCL1-*like might play an important role in inducing the expression of the soybean *DREB* genes.

The expression of *GmDREB2-*like suggested that this gene is only involved during the early responses to drought stress. Under moderate hydration stress, the expression of *GmDREB2-*like was induced (Fig. 2A ), however, a significant reduction in *GmDREB2-*like expression was observed under severe conditions (Fig. 2B). In contrast, *GmDREB1-*like and *GmDREB3-*like gene expression induction was observed under this treatment (Fig. 2B).

The expression of *DREB* genes under cold stress is suppressed upon the expression of PRR transcription factors that also act in the core circadian oscillator [59]. Because significant cross-talk occurs between cold and drought responses, these expression patterns indicate similar regulatory relationships in soybean. The peak expression of the *GmPRR-*like genes corresponds with the reduced expression of the *GmDREB1*-like gene (ZT8) (Fig. 1, 2 and 3). Additionally, the reduced expression of some *GmPRR-*like genes was observed under drought stress (Fig. 1), which is consistent with a mechanism for promoting DREB expression by reducing expression of these repressors.

Cold weather and drought affect plant growth and, ultimately, productivity, and many genes respond to both abiotic stresses at the transcriptional level. The DREB genes act as signaling intermediates for both cold and drought responses, and the promoter region of many *DREB*-induced genes contains a dehydration-responsive element, which is a *cis*-acting element that regulates both cold- and dehydration-responsive gene expression in *Arabidopsis* [71]. There is little definitive information regarding the cross-talk between these two different signal transduction pathways; however, the circadian behavior of the *DREB* genes in response to both cold [59] and drought stresses [70] suggests an association with the circadian clock. The circadian clock functions as a key moderator to coordinate metabolism under stress situations to fine-tune the synchronization of global transcription and physiological processes [11], [29].

We also evaluated the expression of a *GmbZIP1* transcription factor that belongs to the AREB subfamily and is reported to be an abiotic stress- and ABA-responsive gene [72]. The authors suggest that the overexpression of *ZIP1* in *Arabidopsis* and wheat increases drought tolerance and improves the ABA-mediated control of stomatal aperture in plants. The results obtained in the present study show diurnal oscillation for this gene expression in response to moderate stress at dawn (ZT0) compared with the control treatment (Fig. 2A). In addition, the *GmbZIP1* expression was similar to that of the *GmDREB-*like genes, suggesting a putative mechanism involving transcription factors in plant stress defenses that are activated in the early morning before sunlight.

It has been proposed that the *AtABAR* gene functions as an ABA receptor, playing an important role in stomatal closure in response to drought in *Arabidopsis* [10]. Although it was possible to observe diurnal oscillation in the expression of *ABAR* in the present study, we could not detect the upregulation of this gene in response to either drought stress treatment (Fig. 2). The function of ABAR as an ABA receptor has been examined in barley (*Hordeum vulgare* L.), and the authors showed that ABA has no effect on *ABAR/CHLH/GUN5* expression, and ABA binding to the barley protein could not be shown [73].

The *Arabidopsis* ABA signaling system is composed of ABA-bound PYR/RCAR proteins, phosphatases (PP2C) and kinases (SnRK2) and mediates the transmission of the hormone signal [2]. According to this mechanism, ABA binds to PYR/RCAR proteins, releasing SnRK2 from PP2C-induced repression; once activated, the kinases phosphorylate the transcription factors that activate ABA-responsive genes. We observed the upregulation of *GmPP2C-*like and downregulation of *GmSnRK2-*like at the transcriptional level, particularly when comparing the expression levels under severe stress to those under the control conditions (Fig. 2B). The expression of both gene transcripts showed a diurnal oscillation similar to that of *GmDREB-*like and *GmbZIP1-*like in which higher expression levels were observed at predawn hours. The observed regulation of *GmSnRK2*-like and *GmPP2C*-like is consistent with the observations of previous studies on *Vitis vinifera* [74] and *Oryza sativa* [75], respectively.

The *Arabidopsis* orthologs for the *GmRemorin-*like, *GmGols-*like, and *GmRAB18-*like genes used in our study showed diurnal oscillation and ABA upregulation [35]. This is consistent with the results of previous studies of the *Arabidopsis* Remorin ortholog showing the upregulation of gene expression in response to drought [8]. Our results show the upregulation of the *GmRemorin-*like gene under both moderate and severe drought stress (Fig. 2). Interestingly, this gene expression was much higher in response to severe stress and showed a significant diurnal oscillation, with lower expression values between ZT8 and ZT16 (Fig. 2B), whereas the expression differences were not significant under the control and stressed treatments at ZT0. Studies that characterize the function of Remorins are important to understand drought tolerance mechanisms, as Remorins have only been identified in plants [8]. The Remorins have a hydrophilic profile and attach to the plasma membrane. The evolution of the cell wall composition is likely associated with the emergence of different classes of proteins that maintain cell membrane/cell wall integrity and the acquisition of vascular tissue. Thus, it is reasonable to propose that these proteins played an important role during the plant colonization of land in that the chemical adaptation of the cell wall was vital to cope with the particularly rough selection pressure in a dry environment.

The *Gols* gene encodes a key enzyme for raffinose biosynthesis (galactinol synthase), which is an important osmoprotectant associated with defense mechanisms in response to abiotic stresses [6]. The *GmGols-*like expression observed in this study was similar to that of the other drought-induced genes discussed here, showing diurnal oscillation and expression peak just before dawn (Fig. 2 and 3). The *GmGols*-like promoter region also contained an element similar to TBS (Table S2), which is recognized by CHE factors, suggesting a possible interaction between the GmCHE factors and *GmGOLS-*like gene. Additionally, *Arabidopsis AtCHE* has been identified as a putative *AtCCA1* repressor [76], and consistent with this, our data show that the *GmLCL1-*like and *GmGOLS-*like genes have similar expression profiles, with peaks before daybreak (Fig. 1, 2 and 3).

*AtRAB18* is a well-known drought- and ABA-responsive gene used in several studies as a marker for drought and ABA treatments [66], [77], [78]. *RAB18* encodes a protein involved in membrane vesicle transport, and its function has been associated with the recycling of molecules, the removal of existing molecules from cellular compartments, and their replacement with newly synthesized molecules during stress adaptation [7]. The *GmRAB18* gene showed diurnal oscillations under the stress conditions, with higher expression during the day between ZT0 and ZT4, in moderate stress (Fig. 2A and 3), and ZT4 and ZT12 in severe stress (Fig. 2B). The moderate drought stress applied appeared to specifically induce the peak expression of *GmRAB18-*like (Fig. 2A), whereas severe stress caused a significantly higher expression level that was approximately 2000 times greater than that in the control plants (Fig. 2B). The expression profile of the soybean gene suggests that the product of *GmRAB18-*like is important during the day because the moderate stress treatment specifically amplified the daytime expression.

It is important highlight that in the present study we evaluate the diurnal oscillation of drought-responsive genes using plants under simulated field conditions, which means light (14h light/10h dark) and temperature (28°C/20°C) cycles. It was not our intention tell apart the contribution of each factor (circadian clock or environment) to gene expression, but to study their interaction, since in real field conditions such separation does not occur. For example, we could not affirm that the early morning changes in gene expression of drought-responsive genes (*DREB2*-like, *bZIP*-like, *GOLS*-like and others) are exclusively due to light-induction, or due to the circadian clock alone. Previous reports suggest that the circadian clock and the environmental responses contribute to the diurnal molecular responses, suggesting that both components interact to regulate the biological processes in plants [79]. Additionally, as previously discussed, studies about *DREB* genes in Arabidopsis show the circadian oscillation of these genes (in constant light/temperature conditions), with gene expression peak at early morning [28], the same pattern observed in our analysis, making very unlikely the hypothesis that the pattern obtained in our study are only due light changes. Thus, based on our results and on literature evidences, we believe that the expression profiles obtained in our study are the result of the interaction between both circadian clock and environmental cues (light and temperature), which results in the diurnal oscillations.

### 3. ABA treatment influences the expression of drought response genes and some circadian clock genes

The phytohormone ABA is integral for the response to stressful environments and for the regulation of growth and development [80]. To understand the effects of ABA as a drought stress response component in soybean and to link the activity of this phytohormone with the diurnal expression patterns observed under drought stress, selected soybean genes were characterized for their responsiveness to exogenous ABA treatment. Plants grown under the control environmental conditions were sprayed with a solution of 100 mM ABA just after the start of the light period (ZT3), and gene expression was determined at 4 h intervals thereafter, beginning at ZT4. We selected representative ABA- and drought-responsive genes (*GmRAB18-*like, *GmGOLS-*like, *GmABAR-*like, and *GmbZIP*) as well as several circadian clock genes (*GmTOC1-*like, *GmLUX-*like, *GmLCL1-*like, *GmELF4-*like, *GmPRR3-*like, *GmPRR9-*like, and *GmJMJ-*like) for the analyses. All of these genes contain putative ABA-responsive elements in their promoter regions (Tables S1 and S2).

**Figure 4:**
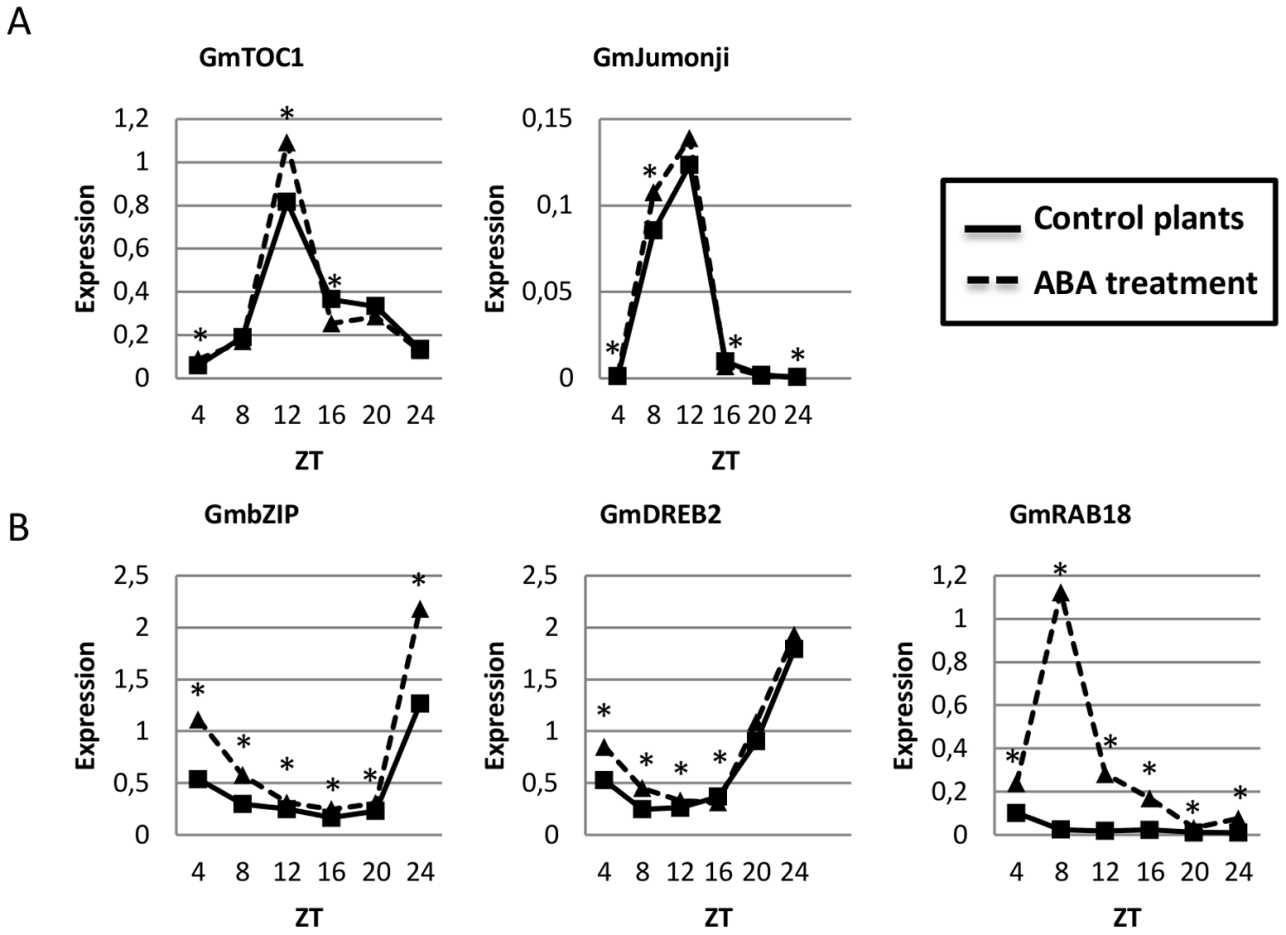
ABA treatment affects the regulation of drought-responsive and some circadian clock genes. Gene expression data regards qPCR analysis of circadian clock (A) and drought-responsive (B) genes. Expression axis represents normalized expression (NE) = 2∧^-(Ct experimental – Ctn)^. Collect time points are represented by ZT (Zeitgeiber Time) 4 to 24, starting 4h after the lights came on ( ZT4) and proceeding with 4 h intervals until ZT24. For easy viewing, asterisks represent significant differences between control and stressed plants in each time point (Duncan’s test 5%, *time -treatment* interaction). The ANOVA and the complete Duncan’s tests results can be found in Table S5.

The candidate ABA- and drought-related genes *GmRAB18*-like, *GmDREB2*-like and *GmbZIP*-like responded to ABA application (Fig. 4; Table S5). On the other hand, *GmABAR-*like, pointed as an ABA receptor for some authors, showed no significant change in its expression levels or patterns following the ABA treatment (Fig. S3). Thus, considering that *GmABAR-*like was nonresponsive to both moderate and severe drought stress (Fig. 2 and 3), it is likely that this soybean homolog is not involved in the ABA-mediated responses to drought.

Although we observed that ABA treatment induced ABA- and drought-related genes under our experimental conditions, we could not detect a strong effect on the circadian clock genes evaluated, with the exception of the evening genes *GmJumonji-like* and *GmTOC1-like* (Fig. 4; Fig.S3; Table S5). The *GmTOC1-*like responses to ABA were of particular interest, as exogenous ABA treatment induces the expression of *Arabidopsis TOC1* [10]. Furthermore, *Arabidopsis* TOC1 participates in a feedback loop with ABAR to modulate ABA responsiveness, a process which is involved in drought tolerance [10]. However *GmTOC1*-like expression showed a significant decrease in response to ABA treatment at ZT16 (Fig. 4A), requiring further investigation of this gene responses to ABA.

### 4. Conserved cis-elements are located upstream of soybean circadian clock and drought-responsive genes

Diurnal oscillation was evident for both soybean clock and drought-responsive genes, suggesting the circadian regulation of these genes. Transcriptional regulation is expected to occur through conserved circadian and drought-responsive *cis-*elements present in the promoter regions of these genes. Therefore, the presumed promoter regions, corresponding to 100 bp located at the 5′ end and the 500 bp located upstream of all 11 circadian clock and 10 drought response genes were analyzed for the presence of known *cis*-elements. Sequence motifs similar to circadian and light *cis*-elements were present in the promoters of several of the drought-responsive genes in our study (Table S2). These elements included potential evening elements (atgaaaAATAtcatc), GAP-box light response elements (taaaATGAagagtag), light-responsive element motifs (tcATCTataca), and TBS elements. These results confirm the gene expression data showing strong diurnal oscillation of the majority of the drought-responsive genes evaluated, and suggests the involvement of the circadian clock in these genes regulation.

In *Arabidopsis*, CCA1/LHY repress *AtTOC1* and *AtLUX* expression by directly binding to the evening element in the promoter regions of these genes [14], [54]. The promoter regions of the *GmTOC1-*like and *GmLUX-*like genes reveal the presence of evening elements (Table S1). This result is consistent with the idea that LCL1 is involved in the direct regulation of these genes. This is further confirmed by the early phase expression of *GmTOC1-*like and *GmLUX-*like as well as the reduction of *GmLCL1-*like expression under severe stress conditions (Fig. 1B).

The promoter regions of the soybean circadian clock genes analyzed in this study (except the *GmPRR7*-like gene) contain the *cis*-elements involved in plant responses to abiotic stresses (Table S1). These elements include the dehydration-responsive elements (the binding site for DREB transcription factors), ethylene response elements (targets of AP2/EREBP transcription factors), salt/drought-responsive elements (targets of such stress-responsive elements as zinc-finger proteins), ABA-responsive elements, ER stress response elements, and heat shock elements (targets of heat shock transcription factors). The presence of these stress-responsive *cis-*elements in circadian clock gene promoters supports the idea of gene expression alterations in response to water deficit, and highlights the connection between the plant stress defenses induced to reduce cellular damage and the perception of the day/night environment, possibly through the circadian clock.

## Conclusion

Our results show that the drought stress affects the gene expression of circadian clock components in soybeans. We also demonstrate the diurnal oscillation of soybean drought-responsive genes expression. This result explains discrepancies in the gene expression data available in the literature, as previously suggested [27], indicating that the daily expression fluctuations are the primary source of variation between independent experiments [27]. Together our results suggest a possible regulatory interaction between the drought responses and the circadian clock genes in soybean. Interestingly we observed that many of the drought-induced genes associated with the plant defense showed expression profiles with higher intensities before/during dawn under our experimental conditions. It is reasonable consider that may exist interaction between the expression of cell dehydration defense genes and the circadian clock genes to optimize plant metabolism to specific periods of the day, since such mechanism could play a key role in increasing survival and reproductive efficiency in arid environments. Characterizing the components of this mechanism will contribute to the development of genetic engineering strategies to improve drought tolerance in plants for desirable agronomic productivity.

## Supporting Information

Figure S1 **Phylogenetic tree for the PRR genes.** The proteins encoded by *Arabidopsis* AtPRR3, AtPRR7, and AtPRR9, the soybean GmPRR homologs, and its paralogs were used to construct the tree using the ClustalW algorithm in the MEGA 5 program. The Neighbor-Joining method was used with the following parameters: Poisson correction, pairwise deletion, and bootstrapping (1000 replicates; random seed).
(PDF)

Figure S2 **Model of the impact of severe drought on circadian clock genes.** Model of the impact of severe drought stress on *GmLCL1*, *GmTOC1*, *GmLUX* and *GmELF4-like* gene expression.
(PDF)

Figure S3 **The circadian clock genes that exhibited no response to ABA treatment.** Gene expression data regards qPCR analysis. Expression axis represents normalized expression (NE) = 2∧^-(Ct experimental – Ctn)^. Collect time points are represented by ZT (Zeitgeiber Time) 4 to 24, starting 4h after the lights came on (ZT4) and proceeding with 4 h intervals until ZT24.
(PDF)

Table S1 **Putative** ***cis*** **-regulatory elements on circadian clock genes.** Putative *cis*-regulatory elements located in the promoter regions of the soybean circadian clock genes.
(XLSX)

Table S2 **Putative** ***cis*** **-regulatory elements on drought-responsive genes.** Putative *cis*-regulatory elements located in the promoter regions of soybean drought-responsive genes.
(XLSX)

Table S3 **ANOVA and Duncan’s multiple range test for multiple comparisons among groups (5%) to evaluate the effects of water regimes (control, moderate and severe drought), time point (ZT0 to ZT20), and their interactions.** Data regards qPCR analyses.
(XLSX)

Table S4 **ANOVA and Tukey’s HSD multiple comparison test (95% family-wise confidence level) to evaluate the effects of water regimes (control and moderate drought), time point (ZT0 to ZT20), and their interactions.** p-values are shown; bold numbers represent significant gene expression differences (p-values ≤0.05). Data regards RNA-seq analyses.
(XLSX)

Table S5 **ANOVA and Duncan’s multiple range test for multiple comparisons among groups (5%) to evaluate the effects of ABA treatment, time point (ZT4 to ZT24), and their interactions. Data regards qPCR analyses.**
(XLSX)
