# Supplementary figures and images for: Diurnal Oscillations of Soybean Circadian Clock and Drought Responsive Genes
Source: PLoS One. 2014 Jan 27;9(1):e86402. doi: 10.1371/journal.pone.0086402 (PMC3903518; doi:10.1371/journal.pone.0086402)

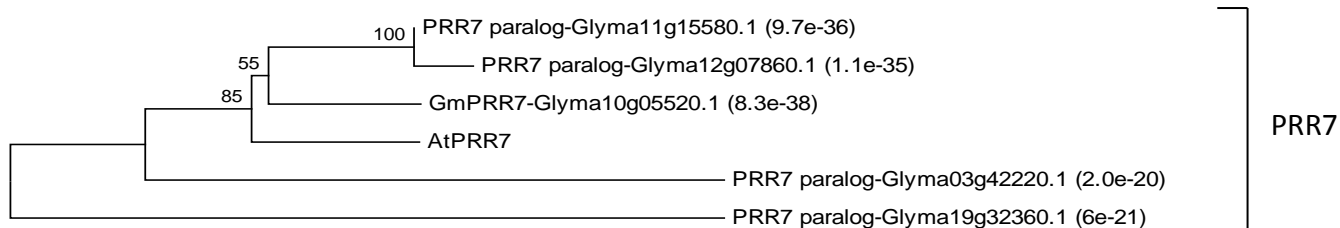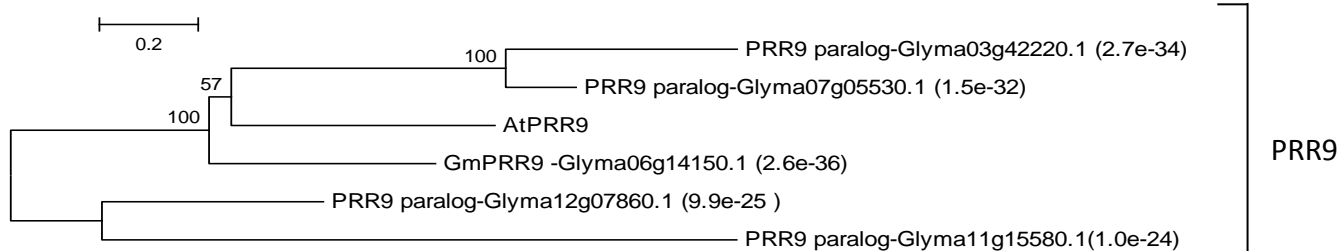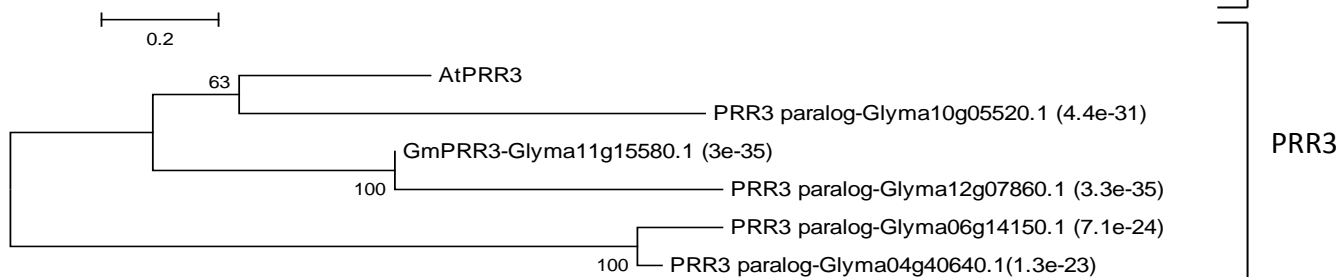

Supplement: Figure S1 — Phylogenetic tree for the PRR genes. The proteins encoded by Arabidopsis AtPRR3, AtPRR7, and AtPRR9, the soybean GmPRR homologs, and its paralogs were used to construct the tree using the ClustalW algorithm in the MEGA 5 program. The Neighbor-Joining method was used with the following parameters: Poisson correction, pairwise deletion, and bootstrapping (1000 replicates; random seed). (PDF) [file pone.0086402.s001.pdf]

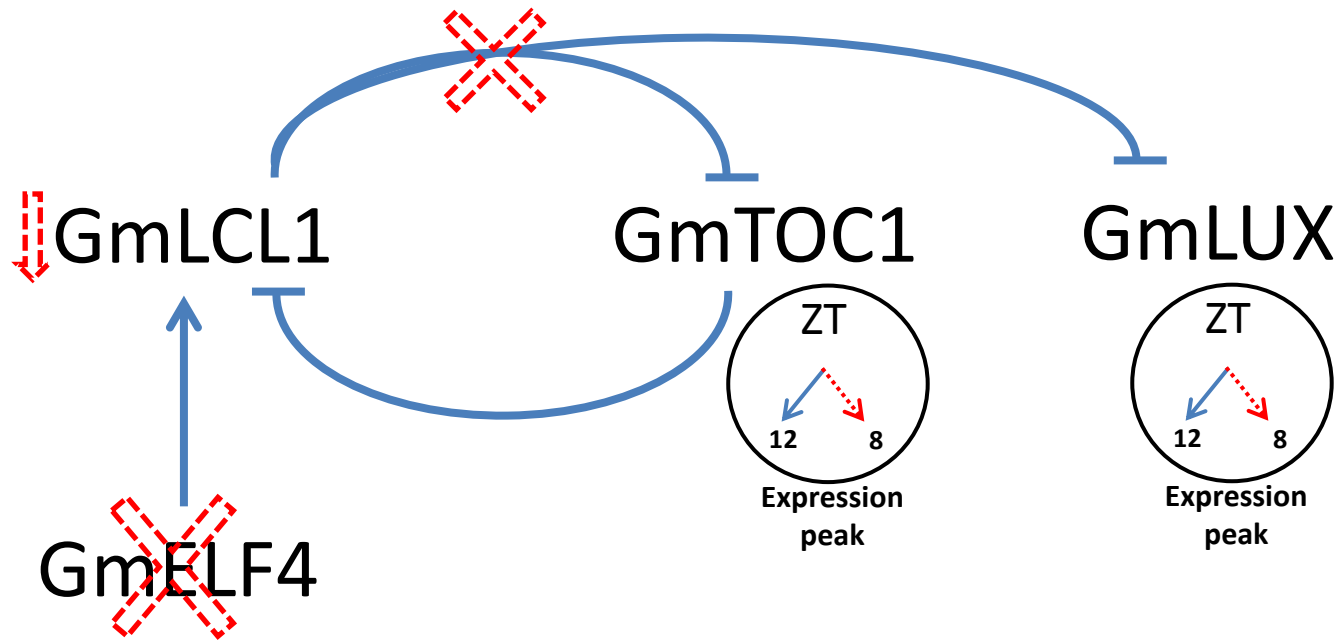

Blue: normal hydration conditions

Red: Severe drought stress

Supplement: Figure S2 — Model of the impact of severe drought on circadian clock genes. Model of the impact of severe drought stress on GmLCL1, GmTOC1, GmLUX and GmELF4-like gene expression. (PDF) [file pone.0086402.s002.pdf]

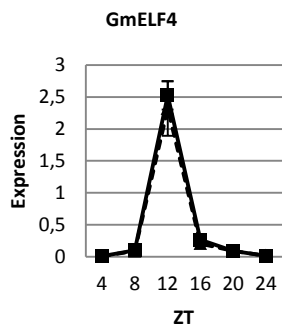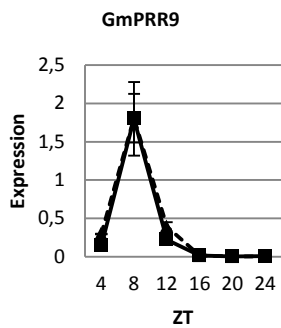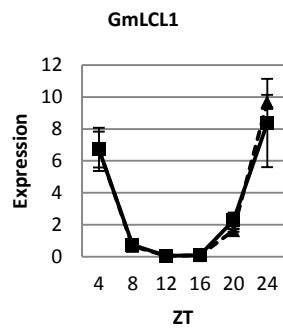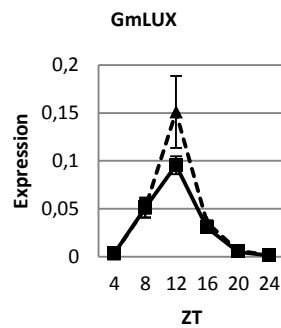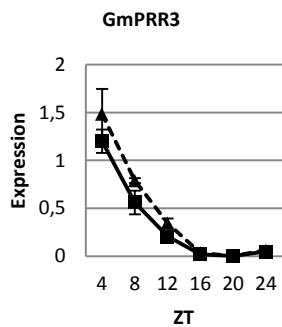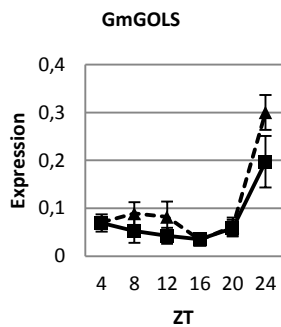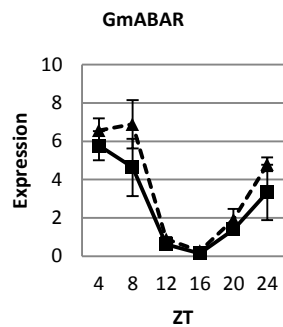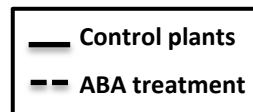

Supplement: Figure S3 — The circadian clock genes that exhibited no response to ABA treatment. Gene expression data regards qPCR analysis. Expression axis represents normalized expression (NE) = 2∧-(Ct experimental – Ctn). Collect time points are represented by ZT (Zeitgeiber Time) 4 to 24, starting 4h after the lights came on (ZT4) and proceeding with 4 h intervals until ZT24. (PDF) [file pone.0086402.s003.pdf]
